# Interpretation of Medical Imaging Data with a Mobile Application: A Mobile Digital Imaging Processing Environment

**DOI:** 10.3389/fneur.2013.00085

**Published:** 2013-07-04

**Authors:** Meng Kuan Lin, Oliver Nicolini, Harald Waxenegger, Graham J. Galloway, Jeremy F. P. Ullmann, Andrew L. Janke

**Affiliations:** ^1^Centre for Advanced Imaging, The University of Queensland, Brisbane, QLD, Australia

**Keywords:** mobile digital imaging processing, mobile visualization tool, medical science, bioimaging, biomedical research, neuroinformatics

## Abstract

Digital Imaging Processing (DIP) requires data extraction and output from a visualization tool to be consistent. Data handling and transmission between the server and a user is a systematic process in service interpretation. The use of integrated medical services for management and viewing of imaging data in combination with a mobile visualization tool can be greatly facilitated by data analysis and interpretation. This paper presents an integrated mobile application and DIP service, called M-DIP. The objective of the system is to (1) automate the direct data tiling, conversion, pre-tiling of brain images from Medical Imaging NetCDF (MINC), Neuroimaging Informatics Technology Initiative (NIFTI) to RAW formats; (2) speed up querying of imaging measurement; and (3) display high-level of images with three dimensions in real world coordinates. In addition, M-DIP provides the ability to work on a mobile or tablet device without any software installation using web-based protocols. M-DIP implements three levels of architecture with a relational middle-layer database, a stand-alone DIP server, and a mobile application logic middle level realizing user interpretation for direct querying and communication. This imaging software has the ability to display biological imaging data at multiple zoom levels and to increase its quality to meet users’ expectations. Interpretation of bioimaging data is facilitated by an interface analogous to online mapping services using real world coordinate browsing. This allows mobile devices to display multiple datasets simultaneously from a remote site. M-DIP can be used as a measurement repository that can be accessed by any network environment, such as a portable mobile or tablet device. In addition, this system and combination with mobile applications are establishing a virtualization tool in the neuroinformatics field to speed interpretation services.

## Introduction

Digital Imaging Processing (DIP) is a rapidly evolving area in medical science. It plays an important role in the analysis, interpretation, and viewing of data. It has been widely introduced due to technological advances in digital imaging, computer processors, and mass storage devices (1). In medical science, DIP involves the modification of data to improve image quality for interpretation. Processing and extraction of information from images is indispensable in the experimental workflow of medical science research, and in particular in cell biology and neuroscience (2). DIP helps not only in maximizing details of interest for image extraction and analysis but also provides the capability of capturing the raw data for further study and interpretation.

New digital acquisition techniques in biomedical research have led to a vast increase of scientific data across spatial and temporal scales (3). Technological improvements in the last decade, such as image resolution, contrast enhancement, and microscopic precision have resulted in the ever-increasing size of data (4, 5). These datasets are multi-gigabyte/terabyte when re-stacked (reconstructing serial sections to a 3D block). This has complicated image manipulation, retrieval, and sharing. Several systems have addressed the issue of image processing and data management on data handling, extraction, protocol management, and real world collaboration such as the LONI system from UCLA, (6) XNAT system, (7) and HID system (8) [for a review, see Jessica and John (9)]. A great challenge for DIP is that the extraction of information from large raw data sets is slow and can limit scientific progress. To overcome these issues, the expertise for developing enterprise software tools or even simply running the hardware necessary for this scale of data management and analysis has been introduced in individual laboratories (2). This processing can still only be done by specialized hardware with advanced processors and mass storage. These features are not conducive to remote viewing due to the need for massive data communication.

In the biomedical field, several open source neuroinformatics implementations of DIP have been introduced (10, 11). For example, an open source cell finding and tracking package called ZFIQ (12) is available for quantitative, reproducible, and accurate data analysis. Those implementations serve well to acknowledge the basic parameters of a biomedical study (13). However, recent advances in mobile technology have increased the application of technology to telemedicine and imaging processing (14). This technology can greatly increase the ability of local hospitals or clinics to provide general neurologic consultation services (15). It is now possible to perform DIP so that users can query images remotely without significant data transfer. Mobile devices that support this must be capable of displaying imaging data (with tri-planar views) and be able to increase performance of direct image tiling.

The purpose of this study is to (a) facilitate a greater mobile DIP application for medical science, (b) offer an innovative application that displays information graphically, (c) increase the performance of data handling, and (d) increase the speed of rendering, particularly for MRI datasets. This study describes a comprehensive design for the current mobile implementation and future potential of remote tools in medical science. The application developed by the authors of this paper is called TissueStack (4). TissueStack is a HTML5 multi-dimensional tile-based image-viewing platform with targeted interfaces for desktop and mobile clients. It allows visualization of high-resolution MRI and histological data. The server component runs on an Apache Tomcat webserver, is multi-threaded and connected to a Postgres (16) database. The development was motivated by the need to view large multi-dimensional MRI and histological imaging data in the neuroinformatics field.

### Software design

Rapid advances in bioimaging technology and mobile networks enable an M-DIP system to provide improvements in the quality of image extraction and workflow speed (17–19). M-DIP for bioimaging services is intended to provide: (3) (a) solid technological architecture; (b) transparent data extraction and querying; (c) technical and semantic interoperability between multiple datasets; (d) demonstrated operational efficiency and flexibility; and (e) Reliable user access and support with a friendly user interface (UI). M-DIP exhibits a comprehensive framework for multi-center data acquisition and is deployed for TissueStack datasets – specifically from Medical Imaging NetCDF (MINC), Neuroimaging Informatics Technology Initiative (NIFTI), or RAW image formats. The aim of using a M-DIP is to select single voxels from TissueStack datasets and perform image interpretation. Three main techniques of data image processing have been implemented in M-DIP: image lookup, data extraction, and direct tiling. The front-end interface is comprised of two components: a *web* and *direct querying* service.

#### System architecture

**Figure 1:**
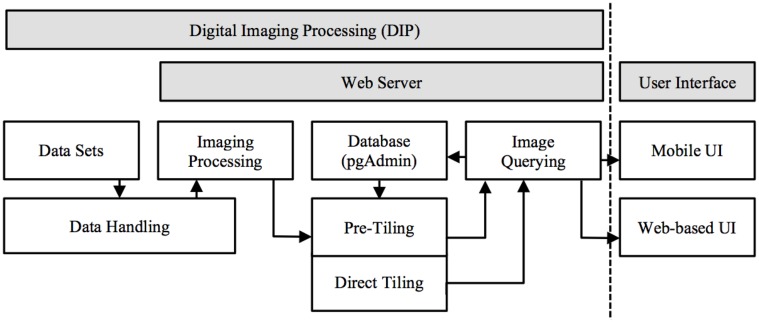
**Mobile digital imaging processing system architecture**. Image processed in server side and presented in front-end UI (data float from left to right and handled based on dataset format).

M-DIP provides a structured information context for histological and MRI voxel data interpretation. It includes a low-level server and high-level client. Several programing languages were used: (a) Structured Query Language (SQL) for relational database based image queries; (b) server side C programing for image processing, data handling, and manipulation; and (c) Java programing (Oracle Inc., USA) for server and client side web services. M-DIP implements a three-tier architecture including a DIP server (back-end server side), an application middle tier with a relational database (middle-end) and a mobile (front-end) UI (Figure 1).

The three-level design can be both modular and flexible depending on the needs of the user. The server side performs three main functions: (a) *data handling and conversion*: the dataset is capable of being loaded into memory to be used by the server or the dataset requires conversion to RAW file format. (b) *Data Manipulation*: extracting from the volume the slice(s) needed, image processing (color mapping, scaling, and rotation), and passing the result to the Server. (c) *Server Communication*: once the data is ready to be sent, the server sends the data via sockets [Transmission Control Protocol (TCP) and UNIX] and can be handled locally (by another program using a UNIX socket) or remotely using a standard TCP connection. A communication component has been created to communicate directly with the browser and send images as binary data.

A Java middle-layer links the database, front-end, and server. It handles new information sent by the server, stores it and retrieves it from the database. Afterward, the information can be transmitted to the front-end interface. To facilitate the communication from the front-end to the middle-end Java, a web service has been created and integrated into the latter. The responses produced are formatted as JavaScript Object Notation (JSON) or Extensible Markup Language (XML). The web service also communicates with the server side through a UNIX socket when the two instances are on the same server and through TCP when one part is remote.

**Figure 2:**
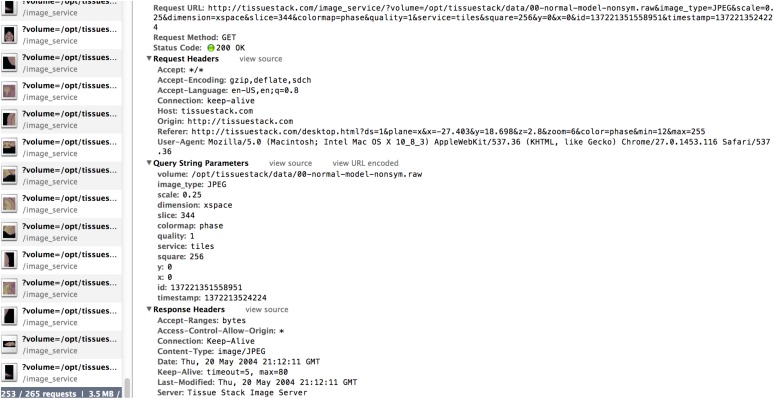
**Example of the RAW file querying request as shown by Google Chrome Webkit Inspector**.

The third part of the system consists of an HTML5 web-based front-end, the UI for the final user. It is the only visible part of TissueStack to a user, nevertheless, this part communicates frequently with other layers via Asynchronous JavaScript and XML (AJAX) requests. Figure 2 shows an example of the image-querying request from Google Chrome webkit inspector. This service ensures a better user experience by increasing responsiveness as the entire page is not reloaded with each request.

#### Data handling (server side)

A major problem with current imaging formats is how data are represented and stored inside a volume. MINC (20) or NIFTI (21) files store only one dimension of data linearly; other dimensions are retrieved via permutation (22). This method severely affects the performance of data retrieval and storage. When accessing the first dimension, speed and resources used are constant. But the remaining dimensions will be progressively slower due to permutation. The storage techniques used by MINC and NIFTI files have their advantages (e.g., file size and portability) but are not conducive to high performance image retrieval. The speed of data retrieval is critical when performing image extraction in real time. Three main solutions exist to overcome this problem: (a) down-sampling the data and creating smaller datasets, (b) dicing the volume into multiple smaller cubes, and (c) loading the entire volume into the memory. Down-sampling increases the performance of data manipulation but the quality loss inherent in this technique is not suitable for this project. Dicing requires the creation of a file and directory structure that is fragile to file movement and can be difficult to maintain. However, it is still the solution used by many imaging tools to improve the general speed of applications (23). Loading the entire file into memory is not feasible because significant RAM is required for Giga/Tera byte datasets. These three solutions were not used in TissueStack and instead we chose to create a specific imaging format that could meet the project’s primary need of speed.

Our specific file format, known as RAW, not only stores data linearly for the first dimension but for all dimensions. This implies redundancy as all voxel values are repeated *x* times where *x* is the number of dimensions. However, this solution is currently only tractable with three-dimensional datasets as adding further dimensions increases the storage space required exponentially. Despite increasing the storage size, some benefits are: (a) this format stores the voxels as a raw value meaning that no modification, transformation or translation is done on the original voxel values. These values can be copied directly into a new file (e.g., jpeg, png.) without computational overhead; (b) the redundancy allows fetching the data linearly for all dimensions. This avoids the need to perform dimension permutation that is undertaken on other imaging formats resulting in a speed loss as a function of volume size; (c) there is no need to memory-load an entire volume as this can be done with portions due to the sequential nature of the file; and (d) it is straightforward to offset and retrieve any data in the volume. Theoretically, the dataset could be infinite and achieve constant performance in fetching any slice/voxel in any dimension of the volume. Despite the *speed* of this file format, the associated files remain simplistic with a minimal header (see Table 1). To ease the adoption of this file format, an automatic converter has been created to support the automatic conversion from MINC and NIFTI to the RAW file format.

**Table 1:** **Header of a RAW file**.

Header format	
@IaMraW@—126—3—499:1311:679—−124.2: −327.15: −169.2—0.5:0.5:0.5—zspace—yspace—xspace—890169:338821:654189—0:444194331:888388662—	
Field/value (in order)	Description
@IaMraW@	RAW identifier
126	Size header
3	Dimension number
499:1311:679	Slices per dimension (e.g., first dimension: 499 slices, second: 1311 slices, third: 679)
−124.2: −327.15: −169.2	World space mm start of each dimension (e.g., first dimension starts at −124.2 mm)
0.5:0.5:0.5	mm Steps for each dimension
zspace—yspace—xspace	Dimension names
890169:338821:654189	Slice sizes (voxels) per dimension (e.g., first dimension: 890169 voxels per slice)
0:444194331:888388662	Offsets of the first slice of each dimension

#### Web access and mobile interface (front-end)

**Figure 3:**
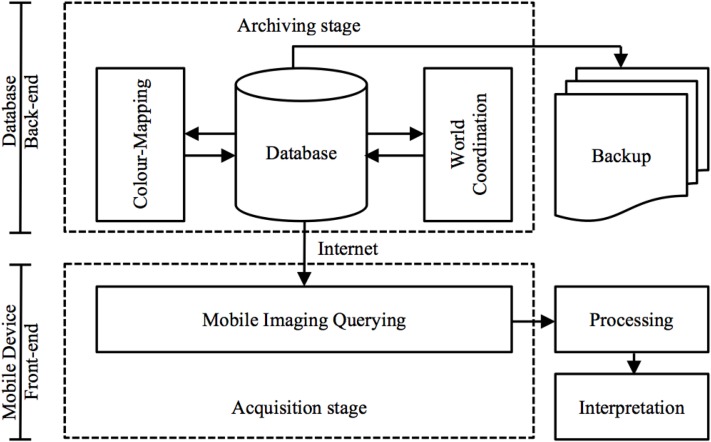
**Data flow via a mobile virtualization tool (application)**. An archiving stage (back-end) focuses on database management and acquisition stages (front-end) handle image-querying and interpretation.

The front-end of M-DIP is divided into five main modules: data and imaging, manipulation, world coordinates, imaging querying, color mapping, and system administration. Screenshots of the mobile UI are in the Section “Results” (Figure 9). This multi-level mobile application features two distinct stages of data flow: (a) an archiving stage with database management and a web access front-end; and (b) an acquisition stages that enables data querying and image interpretation for subsequent analysis (Figure 3).

**Figure 4:**
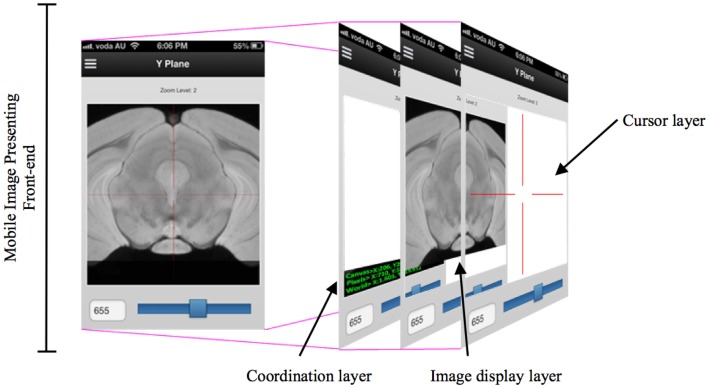
**A multiple layers in a mobile digital imaging processing application**. Layers include but are not limited to a coordination layer, image display layer, and a cursor layer.

While developing the mobile application to cooperate with the DIP server, the various layers of interface need to be implemented so that researchers can access DIP models in a seamless and intuitive fashion and easily access other images in the same plane. When designing a mobile UI, it is useful to provide a familiar style to take advantage of human recognition (24). M-DIP adopts this idea in its development goals. The UI acts in a fashion similar to a mapping tool so that it is intuitive to use. The interface also provides a cross-section tool to zoom in and out for displaying longitudinal planes (Figure 4) so that users can easily interpret histological and MRI data at different scales without image degradation. The M-DIP interface has three layers: a superficial and independent cursor layer that prevents redrawing; a middle-layer that contains the raw images; and a deep layer that contains the world coordinates. Unlike the top layer, the bottom two need to be redrawn following every user interaction.

**Figure 5:**
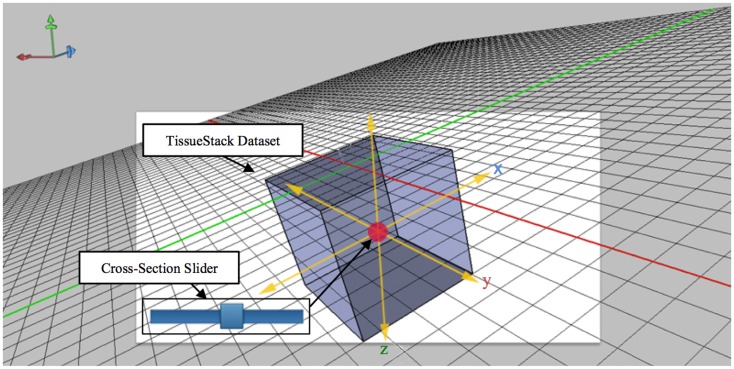
**Cross-section slider for viewing any plane defined by two of the axes along a third axis orthogonal to aforementioned plane**.

The technique shown in Figure 5 extends the idea of an area cursor by allowing users to move the image object as well as coordinates using touch gestures. Users can interpret histological data while querying images by moving their fingers on top of the object, where the relative positions can be associated with other tissue planes. For example, when users interact with the *x* dimension at the point [106,72,52], the *y* dimension will move from the previous position to [52,72,106]. This method of moving the longitudinal plane to interpret data is necessary because it can enable effective search and interpretation of image data without display artifacts. During acquisition, M-DIP can provide enough information for users by querying and applying image processing in the real time such as color mapping.

**Figure 6:**
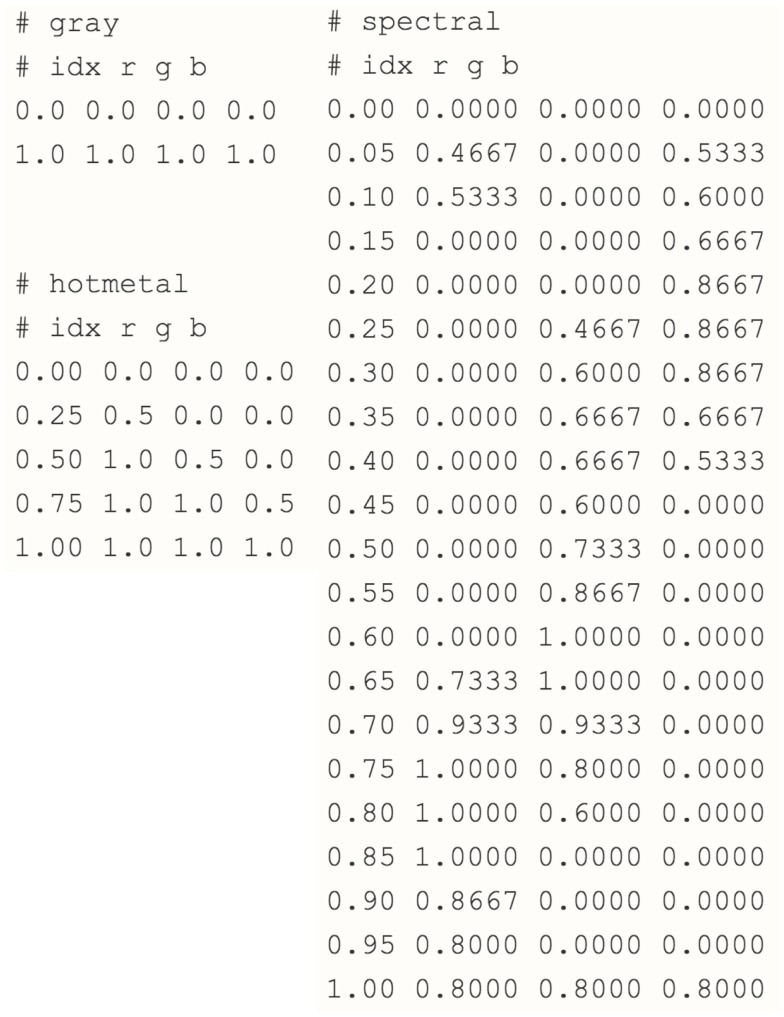
**Gray, Hot Metal, and Spectral color mapping aquarium**.

Remote access via a standard Internet (mobile) connection allows users to extend both metadata and workflow models for analysis. This type of service is referred to as an “archiving stage” where a back-end service holds and manages the data and delivers a view of the images to the front-end interface. The service includes color mapping that displays images in a contrast view and allows cell regions to be tracked and identified in a TissueStack dataset. The key M-DIP implementation in the archiving stage includes the following two components: (a) *World coordinate space:* a key concept for interpretation in the histological field is the spatial representation of the data. The object can be constructed from data on the client side and displayed with respect to a world coordinate space. This millimetric mapping allows users to consistently locate and identify sub-regions in a dataset. Via external registration and realignment regions and co-ordinated can be related between multiple datasets; and (b) *Color mapping:* this is an automated transformation of the image that allows a user to compress or expand the range of image values being displayed. It also allows arbitrary colors to be assigned to different parts of an image based upon their intensity. Figure 6 shows an example of “gray,” “hot metal,” and “spectral” color map RGB values. The first column of each color map represents the gray-scale intensity. The other three represents the RGB values mapped to the first column. All the value goes from 0 to 1. The number of steps (rows) from 0 to 1 defines the complexity of the spectrum of the color map. This allows the presentation of large-scale datasets in a compelling graphical manner.

#### User roles

Based on reported use of DIP in the medical science field (1, 25, 26), M-DIP should allow users to view, annotate, and measure multi-dimensional images remotely. For this paper we focus on a possible solution to DIP in a mobile environment for users in the neuroinformatics field. There are three major users: researchers, editors, and administrators. Researchers use this application for data interpretation; editors can upload, edit, perform image tiling, and conversion on data; administrators have rights to access the image server and database and change user roles. Our implementation embraces new technologies for user interaction and most of this has been conducted in environments where adoption is voluntary. This voluntary use environment is defined as “one in which users perceive the technology adoption or use to be a good choice; a mandated environment is where users perceive use to be organizationally compulsory.” (27) In this user role, the adopted system will be able to complete job tasks that are tightly integrated with multiple users (28).

## Materials and Methods

This research has focused on three key elements (data handling, link-speed, and mobile UI) in this web-based mobile application development for processing medical images, particularly on MRI datasets. (a) *Data handling*: create a specific imaging format that could meet the specialists’ need (increase speed and associated performance) to enhance its information and “system” qualities. (b) *Link-speed*: test system performance and the communication between the mobile and image server to meet specialists’ “satisfaction” and increase “service quality.” (c) *Mobile UI*: provide a mobile graphical interface for visualizing image data to create an environment for image interpretation and analysis in the real time.

To improve the quality of our M-DIP implementation we refer to the overall level of user need, and also use it as a metric to measure information, system, and service qualities. Our implementation will achieve these qualities by providing new methods of data handling with both theoretical and practical contributions. To demonstrate the effectiveness of the TissueStack we have evaluated the system using the Information System Success Model (ISSM) (29). The ISSM states clear, specific dimensions of success, or acceptance for a new system implementation and the relationships between users. In the ISSM, “intention to use” or “user satisfaction” is determined by “information,” “system,” and “service” qualities (30). By combining these independent factors, we used a survey in order to identify users’ experience and intention while using M-DIP.

### Data handling

To demonstrate the benefit of a RAW file over a MINC/NIFTI file, we performed a speed test of extraction of all slices from all dimensions of a dataset. We calculated the number of “operation per seconds” (OPS) for a volume using the formula:

OPS=“number of slices”/“time spent to retrieve all the slices”

In addition, we compared the MINC/NIFTI and RAW file performances in this specific environment with a measurement of OPS. For instance, a simple ratio between the RAW OPS and the MINC OPS of the same volume could quantify a speed gain; however, the OPS are dependent on the file size. Therefore, the file size of the dataset needs to be considered to obtain an accurate representation of numerical gain. The gain is calculated with the following formula:

$$\begin{array}{llll} \text{Gai}{\text{n}}^{\text{M}}= & \left(\text{“MINC file size”/MINC OPS}\right)/\; & & \;\\ & \left(\text{“RAW file size”/RAW OPS}\right)\; & & \;\\ \text{Gai}{\text{n}}^{\text{N}}= & \left(\text{“NIFTI file size”/NIFTI OPS}\right)/\; & & \;\\ & \left(\text{“RAW file size”/RAW OPS}\right)\; & & \; \end{array}$$

### Link-speed

To demonstrate the effect of link-speed on the user experience, a “network degradation” test was performed. Instances of TissueStack were loaded with different network speeds for two datasets and the respective rendering times (RT) recorded. RT was calculated with the following formula:

RT =“time when TissueStack fully loaded” - “time requested”

This RT formula illustrates the time difference while querying different size of datasets. The evaluation is to perform service and information qualities while using mobile devices for bioimaging interpretation.

### Mobile user interface

A number of software development methods were used while creating the mobile interface in order to maximize user image interpretation and analysis. These methods were chosen to ensure “information quality” in the resulting interface.

The first was to take special perceptual aspects into account so that this implementation can be used with minimal errors and frustration. Secondly, the mobile UI will provide content adaptation and prioritization of the most relevant items when a user searches for information. This is referred to “adaptation of visible components layout.” (31) Thirdly, all implementation followed the principle of “keeps it super simple (KISS).” (32) M-DIP aims to change the way bioimaging is presented and increase users “intention to use” and “user satisfaction.”

## Results

**Figure 7:**
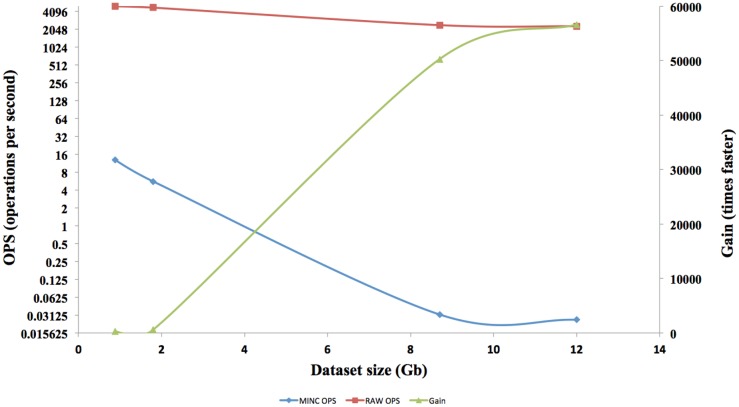
**Operation per seconds and Gain speed comparing MINC and Raw files**.

The interface response times of four different size datasets in RAW format are shown in Figure 7. Each of the speed tests was repeated for MINC files. Afterward, the results are compared and a gain in term of speed is calculated. The speed gain is plotted on the right axis and speed on the left axis.

**Figure 8:**
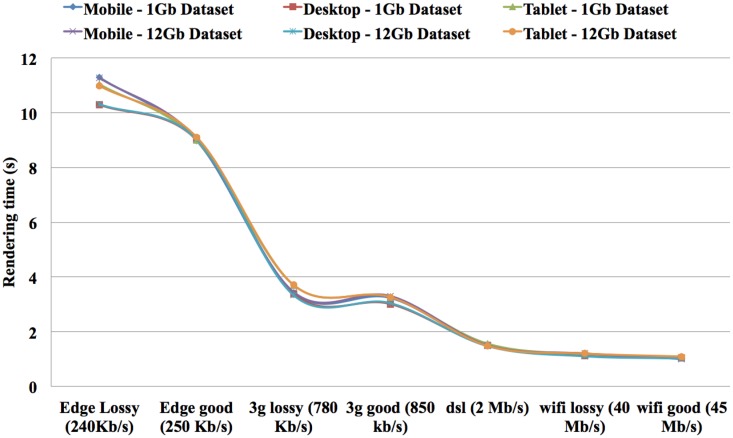
**Rendering time compared to link-speed and dataset size from different devices**.

The speed results show that there is a significant gain in speed when using a RAW file and this speed increase improves as the dataset size increases. The RAW’s OPS also reduced slightly when the volume size increased but not on the scale that a MINC file does. The RT for RAW files from 1GB to 12GB on both the TissueStack mobile (M-DIP) and desktop platforms (DIP) while varying the link-speed is shown in Figure 8.

As expected, the RT reduces when the connection speed increases. Importantly, RT is only affected by the connection speed and not by the volume size. For example, it took 1 s to display a ∼12 GB dataset on a mobile device with a 45 Mb/s link-speed while it took 11.28 s to display same dataset with 240 kb/s connection. In contrast, it took 1.03 s to display ∼1 GB dataset in mobile device with 45 Mb/s link-speed while it took 11.30 s to display same dataset with 240 kb/s connection. This shows that the RT can be considered equal on both the mobile and desktop platforms. The results show that it took 1.20 s to display ∼12 GB dataset in tablet device with 40 Mb/s link-speed while it took 1.1 s to display same dataset in a same link-speed condition in a desktop device. In addition, it took 1.18 s to display ∼1 GB dataset in tablet device with 40 Mb/s link-speed while it took 1.12 s to display same dataset in a same link-speed condition in a desktop device. The results show an increase of “service quality” for data communication.

**Figure 9:**
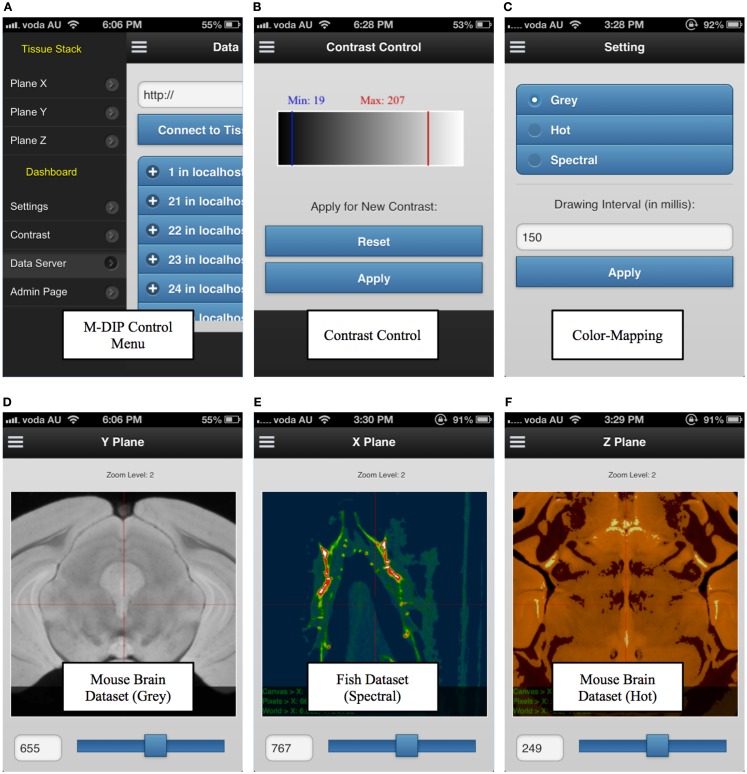
**Mobile digital imaging processing (M-DIP) application user interface**. The user interface contains a control menu **(A)**, a contrast control panel **(B)**, color mapping panel with three choices **(C)**, and the three viewing planes **(D–F)**. In each plane, the slice number and zoom level are also presented.

M-DIP has also been successfully implemented on mobile devices. This M-DIP system has been publicly released for ∼100 tests from 10 specialists who have significant experience using image-viewing software. Each test lasted at least 250 requests, consisting of multiple modality querying (e.g., plain dimensions, color mapping) and behavioral testing for mobile interactions (KISS method). M-DIP is able to query data of up to ∼12TB and process data with the same speed (RT) as desktop browsers with far greater memory and processing power. The M-DIP interface allows researchers and specialists to execute and query in a simple and intuitive manner (see Figure 9), with easy browsing and data interaction. This has been achieved by basing as much as possible of the interface upon known mobile interaction paradigms from existing well known applications such as mobile mapping. Screenshots of various parts of the system are shown in Figure 9 and a demonstration mobile interface can be viewed at http://tissuestack.com/phone.html.

M-DIP has been tested extensively on both iOS and Android devices. These two platforms cover 87.8% of the mobile market in Q4 2012 (33). M-DIP is able to build an atlas view of a larger image, similar to the Google Map application in mobile devices. Overall, M-DIP currently provides a framework for more than 12 unique dataset profiles, with volumes for over 450,000,000 voxels (499 × 1311 × 679). It is able to handle more than three concurrent interactive functions (color mapping, contrast, and zoom levels) and over ∼12TB of imaging data. M-DIP resulted in a highly positive perspective for interpretation potential under mobile environment.

## Discussion

This study addresses the research needs of biomedical researchers and details the specific implications for theory and practice. A good user experience is one of the main goals of any successful application (30, 34). To achieve this, an application must focus on many details such as UI, accessibility, and response time. For a web-based mobile application, a crucial point is RT. Many optimizations can be performed to improve the RT of an application, however at some point it will become dependent on the link-speed and to some extent the dataset size. In our implementation the use of RAW files has reduced this dependence on file size to a negligible amount. Correlating mobile interpretation requirements with a particular service in MRI datasets has led to the mobile application becoming a valuable analysis and diagnosis tool. Our mobile implementation has the ability to retrieve datasets in real time without image degradation even when multiple M-DIP capabilities are used. It is important to note that the use of an M-DIP system without acceptable speed may lead to lower “satisfaction” levels in users’ attitudes toward using the application.

There are numerous products that are capable of digital processing and even managing large-scale three-dimensional datasets on the front-end or the server side. To our knowledge none of these systems have been designed to tackle the speed of data handling in a mobile Internet environment. Although REDCap (35) and Deduce (36) have provided web-based applications for inputting and modifying bioimaging data, there is still no such program that has the ability to increase speed of direct tiling or capabilities for imaging modalities.

In M-DIP, the data access speed has been increased by providing a better real time pre-tiling on the server side. This achievement has been made possible by creating and using RAW files, which provide a considerable speed gain compared to a standard MINC or NIFTI file. The gain generated by using RAW files was higher than expected and was in fact 56,000 times faster than a MINC file. This gain has been made possible due to the RAW file structure and the number of OPS reached. However, the gain cannot be extrapolated above files around 12TB, as even if the RAW OPS were constant, there are still hardware factors [disk Input and Output (I/O)] that would decrease OPS as the volume size increases.

Theoretically, this result should be linear but the system is not perfect and hits hardware limitations such as I/O saturation. Nevertheless, RAW file performance is only half for a 12 times larger volume. Meanwhile, MINC file performance dropped by almost 500 times. RAW files have been created to solve a speed bottleneck for real time tiling, but do not offer all the capabilities of a structured file such as MINC or NIFTI and can therefore only be used for TissueStack’s specific purpose. They are a good solution to meet the speed requirements that determine “system quality” in M-DIP. This method allows the retrieval of data with the shortest time possible and improves the “satisfaction” to users. In addition, we found that dataset size does not hinder the speed of the application by using a RAW file, therefore, it meets the current specialists need and will thus increase their intention to use the application. In addition, RT is only affected by the connection speed and not by the volume size. All versions (mobile, tablet, and desktop) of TissueStack have near identical RT, which also helps the final user experience.

This study demonstrated that the delivery of M-DIP via a specialized format and integrated web-based mobile environment is an efficient tool for imaging specialists. Our survey found that 86% of participants felt that M-DIP was beneficial for initiating appropriate analyses while also making more accurate assessments. Moreover, 98% of participants stated that the M-DIP application facilitated image data visualization. In general, imaging specialists found that M-DIP performed significantly faster in bioimaging data processing than compared to their preferred imaging software and DIP allowed parallel browsing of linked datasets of differing scales. These findings reiterate the development of M-DIP purpose that adopting mobile technology and increasing performance of data handling in DIP are able to deliver better image processing solution to users.

## Conflict of Interest Statement

The authors declare that the research was conducted in the absence of any commercial or financial relationships that could be construed as a potential conflict of interest.
